# Factors influencing health-related quality of life after total hip replacement - a comparison of data from the Swedish and Danish hip arthroplasty registers

**DOI:** 10.1186/1471-2474-14-316

**Published:** 2013-11-06

**Authors:** Max Gordon, Aksel Paulsen, Søren Overgaard, Göran Garellick, Alma B Pedersen, Ola Rolfson

**Affiliations:** 1 Swedish Hip Arthroplasty Register, Gothenburg, Sweden; 2 Division of Orthopaedics, Department of Clinical Sciences at Danderyd Hospital, Karolinska Institute, Danderyds Sjukhus, Sweden; 3 Department of Orthopaedic Surgery and Traumatology, Odense University Hospital, University of Southern Denmark, Odense, Denmark; 4 Danish Hip Arthroplasty Register, Odense, Denmark; 5 Department of Orthopaedics, Institute of Clinical Sciences, The Sahlgrenska Academy, University of Gothenburg, Gothenburg, Sweden; 6 Department of Clinical Epidemiology, Aarhus University Hospital, Aarhus, Denmark

**Keywords:** Total hip replacement, EQ-5D, Predictors, Comorbidity, Patient-reported outcome, Patient-reported outcome measures, Register study

## Abstract

**Background:**

There is an increasing focus on measuring patient-reported outcomes (PROs) as part of routine medical practice, particularly in fields such as joint replacement surgery where pain relief and improvement in health-related quality of life (HRQoL) are primary outcomes. Between-country comparisons of PROs may present difficulties due to cultural differences and differences in the provision of health care. However, in order to understand how these differences affect PROs, common predictors for poor and good outcomes need to be investigated. This cross-sectional study investigates factors influencing health-related quality of life (HRQoL) one year after total hip replacement (THR) surgery in Sweden and in Denmark.

**Methods:**

Data was retrieved from the Swedish (n = 14 560 patients) and Danish (n = 632 patients) Hip Arthroplasty Registers according to preset selection criteria. Using linear regression models, we examined how sex, age, comorbidity and country of surgery were associated with different aspects of HRQoL as measured by the EQ-5D index and EQ VAS.

**Results:**

Danish patients had an overall higher EQ-5D index and EQ VAS than Swedish patients (p < 0.001). After regression analysis, the estimated coefficients for sex, age, or the Charlson score did not differ between countries for either the EQ-5D index (p = 0.83) or EQ VAS (p = 0.41) one year after THR.

**Conclusions:**

We conclude that there are clear similarities in how basic predictors influence patient-reported outcomes (PROs) in patients with THR in Sweden and Denmark and these known predictors of good or poor HRQoL outcomes are not specific for each country.

## Background

There is an increasing focus on measuring patient-reported outcomes (PROs) as part of routine medical practice, particularly in fields such as joint replacement surgery where pain relief and improvement in health-related quality of life (HRQoL) are primary outcomes. Implant survival, radiographic success and absence of adverse events do not guarantee good results from a patient’s perspective. A commonly used instrument to measure patient-reported health outcomes is the EQ-5D, introduced by the EuroQol group in 1987. Health outcomes according to EQ-5D have been found to differ between European countries, calling for caution when making international comparisons of disease burden and health care effectiveness [1]. However, the differences in the EQ-5D index have been found to be rather small regardless of valuation method. Despite between-country variations in the EQ-5D index, the associations between age and sex and EQ-5D index was the same for all countries [2].

National registry data play an essential role in monitoring, developing and improving joint replacement surgery [3]. PRO measures are routinely collected for all patients undergoing total hip replacement (THR) in Sweden by the Swedish Hip Arthroplasty Register (SHAR) [4]. In Denmark, occasional cross-sectional collections of PROMs have been accomplished by the Danish Hip Arthroplasty Register (DHR) in random samples of the Danish THR population. Close collaboration through the Nordic Arthroplasty Register Association allows not only for pooling and comparisons of national implant survival data, but also for analyses of PROs.

The objective of this study was to investigate factors predicting the level of HRQoL one year after THR in patients operated on in Denmark and Sweden. In particular we examined how sex, age, comorbidity and country of surgery were associated with the EQ-5D index and EQ VAS after surgery. We also investigated the external validity of the results by comparing the associated variables between the two countries.

## Methods

### Study population

SHAR, started in 1979, and DHR, started in 1995, gather prospective observational nationwide data. All public and private orthopaedic departments performing THR in Sweden and Denmark report to the respective register. As part of a routine follow-up program, SHAR has been collecting EQ-5D data since 2002. DHR has collected EQ-5D data from a random subset of patients operated in 1999, 2004 and 2008 [5].

For these analyses we selected THRs due to primary osteoarthritis. From SHAR all patients operated in 2006 and 2007 and from DHR all patients operated in 2008 with complete one year EQ-5D data were included. Patients re-operated within a year, or with missing values in any of the outcome scores were excluded from the analysis. For bilateral cases the first operation was selected.

Comorbidity profiles were estimated by cross-matching data with the Swedish National Patient Register and the Danish National Registry of Patients. Both Sweden and Denmark have unique personal identification numbers allowing a perfect match between registers. The disparity in the choice of year for surgery was due to lack of comorbidity data from the Swedish National Patient Register at the time the databases were merged. With these selection criteria we identified 14 560 Swedish patients and 632 Danish patients.

### Outcomes variables

Patients selected for this study had completed a one year follow-up EQ-5D questionnaire sent via ordinary mail. The EQ-5D evaluates subjects in five HRQoL dimensions, namely mobility, self-care, usual activities, pain/discomfort and anxiety/depression [6,7]. In this version of the EQ-5D instrument (EQ-5D-3L) each dimension is divided into three levels of graded severity generating 243 possible response combinations. The EQ-5D can be presented as a global health index with a weighted total value for HRQoL. Due to the lack of a specific Swedish EQ-5D tariff the Danish TTO tariff [8] was used for all patients, ranging from -0.624 to 1, where 1 represents the best possible health state. The EQ-5D also contains a health state visual analogue scale (EQ VAS) ranging from 0, worst imaginable health state, to 100, best imaginable health state.

The primary outcomes were the EQ-5D index and EQ VAS one year after THR. Median time from surgery to follow-up was 1.1 years (range 0.9-1.9) for the Swedish population and 1.2 years (range 0.9-1.5) for the Danish population.

### Independent variables

As independent possible predictors we investigated country, age at surgery, sex, and comorbidity as measured by the Charlson comorbidity score. The Charlson comorbidity score is a prospectively applicable method for classifying co-morbid conditions which might alter the risk of mortality. The Charlson score has 17 disease categories, selected and weighted on the basis of the strength of their association with mortality [9]. It is readily applicable in registry research and we used ICD-10 codes (International Classification of Diseases version 10) for score estimation [10] from hospitalizations one year prior to surgery (including surgery hospitalization). Patients were categorized by three levels: low (no recorded previous diseases), medium (1–2 points) and high (>2 points).

### Statistical methods

We used linear regression models to study the association between independent predictors and the dependent variables EQ-5D index and EQ VAS score. The regression coefficients were calculated with 95% confidence intervals, which were used to describe statistical significance of association. Age was investigated by a restricted cubic spline to avoid residual confounding for the comorbidity variables. The number of knots on the spline curve was chosen by selecting the model with the lowest Akaike information criterion [11]. Effect modification for country was evaluated by ANOVA (analysis of variance).

All analyses where performed using R version 3.0.1, and the rms package (v 4.0-0). Due to non-normal distributed outcomes with heteroscedasticity the confidence intervals were calculated by using a robust covariance matrix (HC3) [12,13].

### Ethical approval

Ethical review approval was obtained from the Central Ethical Review Board in Gothenburg for the merger of the SHAR with the Swedish National Patient Register (decision 328–08) databases, and by amendment the merging of the populations from SHAR and DHR (decision T210-12). The merger of the DHR and Danish National Registry of Patients was approved by the Danish Data Protection Agency (2010-41-5103 and 2013-41-1445).

## Results

### Demography

As presented in Table 1, the demography and comorbidity burden was similar in the two populations. There were no or minor disparities in the demography and comorbidity burden compared to the THR populations with primary osteoarthritis during corresponding time periods (Table 1).

**Table 1:** Characteristics of Swedish and Danish patients

**Variable**	**Denmark**	**Sweden**	**Danish reference THR population**	**Swedish reference THR population**
Male sex	40.5% (256)	42.2% (6148)	43.8% (2579)	43.1% (10 182)
Age	68.5 (± 10.1)	69.4 (± 9.6)	69.1 (± 9.8)	68.6 (± 10.3)
Right side	54.6% (345)	56.1% (8174)	53.3% (3162)	55.5% (13 102)
Year of surgery	2008.3 (± 0.2)	2007.0 (± 0.6)	(2008)	(2006–2007)
Charlson score				
Low	91.0% (575)	86.3% (12 561)	89.7% (5318)	86.8% (20 481)
Medium (1–2)	8.4% (53)	12.8% (1859)	9.4% (557)	12.2% (2888)
High (> 2)	0.6% (4)	1.0% (140)	1.0% (56)	1.0% (249)
Outcomes				
EQ-5D	0.85 (± 0.19)	0.81 (± 0.19)	-	-
EQ-5D VAS	81.6 (± 19.5)	75.5 (± 20.4)	-	-

Data are presented as percentages (number of patients) for proportional variables, while continuous variables are mean ±SD.

### Main results - comparison between countries

**Figure 1:**
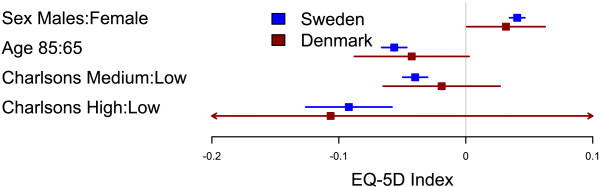
**Comparison of factors influencing EQ-5D index between Swedish and Danish patients.** Forest plot with 95% confidence intervals for the estimates of EQ-5D index one year after THR for gender (reference=female), age 85 years (reference=65 years), and medium or high Charlson (reference=low Charlson) for Swedish (blue) and Danish (red) patients.

**Figure 2:**
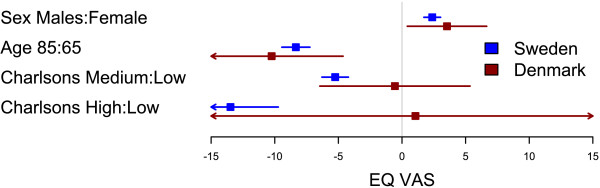
**Comparison of factors influencing EQ VAS between Swedish and Danish patients.** Forest plot with 95% confidence intervals for the estimates of EQ VAS one year after THR for gender (reference=female), age 85 years (reference=65 years), and medium or high Charlson (reference=low Charlson) for Swedish (blue) and Danish (red) patients.

Danish patients had an overall higher EQ-5D index and EQ VAS than Swedish patients (p < 0.001). The distribution of answers in the five EQ-5D dimensions significantly (all p < 0.001) differed between countries; Danish patients scored higher for the mobility, pain/discomfort, and anxiety/depression qualities, while Danish patients scored lower in the usual activities dimension (Table 2). There was no difference between the countries in how sex, age or the Charlson comorbidity index affected the EQ-5D index (p = 0.83) or EQ VAS (p = 0.41) one year after hip replacement (Figures 1 and 2).

**Table 2:** Comparison of EQ-5D dimensions between Swedish and Danish patients

**Dimensions**	**Denmark**	**Sweden**	**P-value**
Mobility			
No problems	79% (502)	59% (8603)	< 0.001
Some problems	20% (129)	41% (5934)	
Confined to bed	0% (1)	0% (20)	
Self-care			
No problems	88% (553)	91% (13 273)	0.0065
Some problems	12% (74)	8% (1187)	
Unable	1% (5)	1% (96)	
Usual activities			
No problems	63% (397)	76% (10 997)	< 0.001
Some problems	34% (212)	22% (3202)	
Unable	4% (23)	2% (360)	
Pain/discomfort			
None	64% (406)	43% (6224)	< 0.001
Moderate	33% (208)	53% (7662)	
Extreme	3% (18)	5% (669)	
Anxiety/depression			
None	87% (549)	77% (11 200)	< 0.001
Moderate	11% (71)	22% (3156)	
Extreme	2% (12)	1% (199)	

Data are presented as percentages (number of patients) and p-values are derived from Fischer’s exact test.

### Other results - identification of overall predictors for PRO

**Figure 3:**
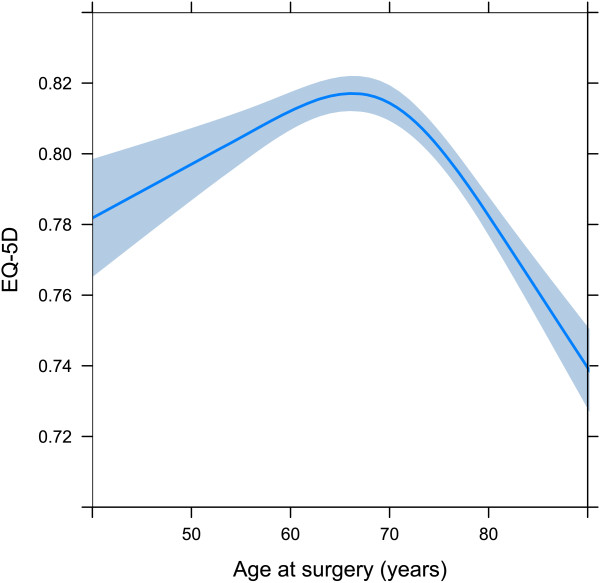
**The age as a spline for EQ-5D index.** The spline is adjusted for sex = female, Charlson score = low, and country = Sweden.

**Figure 4:**
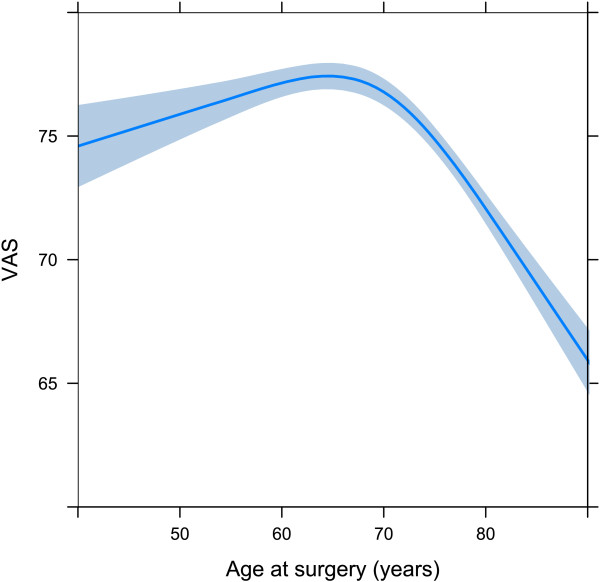
**The age as a spline for EQ VAS.** The spline is adjusted for sex = female, Charlson score = low, and country = Sweden.

Male patients reported higher EQ-5D indexes and EQ VAS scores than females. Patients with a medium or high Charlson comorbidity index at the time of primary THR surgery reported poorer outcomes for the EQ-5D index and EQ VAS compared to patients with low Charlson comorbidity index (Tables 3 and 4). Age behaved in a non-linear pattern, peaking at around 65 years (Figures 3 and 4).

**Table 3:** Association between possible independent predictors and the mean value of EQ-5D index

**Variable**	**Crude**		**Adjusted**	
	**Coef**	**2.5% to 97.5%**	**Coef**	**2.5% to 97.5%**
Intercept	0.815	0.812 to 0.818	0.800	0.759 to 0.841
Sex				
Male	0	ref	0	ref
Female	-0.042	-0.048 to -0.036	-0.040	-0.046 to -0.034
Charlson’s index				
Low	0	ref	0	ref
Medium (1–2)	-0.043	-0.052 to -0.034	-0.039	-0.048 to -0.030
High (> 2)	-0.093	-0.123 to -0.062	-0.092	-0.123 to -0.062
Denmark				
Country = Sweden	-0.041	-0.056 to -0.026	-0.039	-0.054 to -0.024

Adjusted results refer to the full model with all predictors in the table and the spline for age.

**Table 4:** Association between possible independent predictors and the mean value of EQ VAS

**Variable**	**Crude**		**Adjusted**	
	**Coef**	**2.5% to 97.5%**	**Coef**	**2.5% to 97.5%**
Intercept	75.7	75.4 to 76.0	77.5	72.9 to 82.1
Sex				
Male	0	ref	0	ref
Female	-2.7	-3.4 to -2.1	-2.4	-3.1 to -1.8
Charlson’s index				
Low	0	ref	0	ref
Medium (1–2)	-5.8	-6.8 to -4.8	-5.1	-6.1 to -4.1
High (> 2)	-13.8	-17.1 to -10.5	-13.1	-16.8 to -9.3
Denmark				
Country = Sweden	-6.1	-7.7 to -4.4	-5.7	-7.2 to -4.1

Adjusted results refer to the full model with the predictors in the table and the spline for age.

## Discussion

In this study of patients with THR in Denmark and Sweden, we found no significant differences in how age, sex and comorbidity status influence the level of HRQoL one year after surgery. The consistency of these common and basic predictors facilitates pooling and comparisons of data and interpretation of results from the two countries.

### Comparisons between countries

Continual development in joint replacement surgery, continual progresses in medicine, and longer life expectancy, have led to increasing demands for joint replacements. Today there are numerous long-term reports documenting the outstanding survival of many implant designs. One fundamental prerequisite for understanding how to meet the changes in demands on joint replacement surgery is to measure outcomes from a patient perspective. PROMs are becoming increasingly important in the allocation of health care resources and the provision of guidelines for optimum care and management [14]. Large variations between and within countries in disease severity when deciding to operate have been reported [15]. How these variations affect the PROs is sparsely explored. Therefore, in order to compare PROs between countries, these common covariates need to be investigated and compared.

Why Danish patients report higher HRQoL one year postoperatively than Swedish is a question requiring consideration. Firstly, population studies have shown that Danish people in general perceive their HRQoL to be higher than Swedes do [16,17]. Thus, the assumption that EQ-5D values for Danish patients at any time-point are higher than for Swedish patients, would partly explain the observed difference. Secondly, differences in fixation method, implants used and surgical technique may play a role for the PROs. Exempli gratia, the posterior approach is more common in Denmark and previous work indicates that a posterior approach is associated with better PROs as compared to the direct lateral approach [18,19]. Thirdly, the difference in mean age at surgery indicates that Danish patients have surgery at an earlier stage of hip disease. Although age is included in the regression model, there may be residual confounding due to age being a proxy for stage of hip disease. Surgery at an earlier stage of disease implies a preservation of HRQoL after hip replacement. Conversely, surgery at a late stage of disease may reduce the possibility of reaching the expected level of HRQoL for the particular age group. We do not believe the difference in recruitment years explains the observed difference. This view is supported by the consistency in the EQ-5D index over time in the Swedish THR population.

### Age as a predictor

The one-year level of EQ-5D index peaks at about 65 to 70 years. The gradual decline in ages above 70 could be explained by the overall age-dependent abatement seen in the general population. However, the youngest patients did not reach the expected level of EQ-5D. Our interpretation is that younger patients have more active lifestyles and are more likely to be hampered by the limitations of their hip disease, and subsequently their artificial joint, than older patients. The lower mean EQ-5D index in younger patients suggests room for improvement in the way these patients are managed. In order to preserve as much HRQoL as possible among younger patients, persevering with non-surgical treatment options is important. However, it might reasonably be argued that they should have surgery at an earlier stage, and yet the risk of revision complicates this analysis.

### Methodological considerations

The large study population combining a nationwide Swedish THR population and a randomly selected nationwide Danish THR population contributes to the strength of this study. Reoperations have been excluded which limits the effect of potential confounders related to implant survival.

The cross-sectional nature of the study limits comparisons of outcomes between the two countries and few variables have been included in the models. Preoperative PROMs were not available for the Danish patients and thus comparisons taking baseline levels into account were not possible. Furthermore, the Danish population was much smaller than the Swedish and chance may affect Danish data to a greater extent than Swedish. Including a larger Danish population may have resulted in significant differences between countries in how the investigated predictors affect the outcomes. However, the clinical relevance of such small differences would, in that case, be questioned.

## Conclusions

There are clear similarities in how basic predictors influence patient-reported outcomes in patients with THR in Sweden and Denmark. Apparent cultural, social and other such differences among these countries are not reflected in these predictors. However, Danish THR patients exhibit a significantly higher HRQoL one year after surgery compared to Swedish patients. This difference will be subject to further analyses of prospective data.

## Abbreviations

DHR: Danish Hip Arthroplasty Register; HRQoL: Health-Related Quality of Life; PRO: Patient-Reported Outcome; PROM: Patient-Reported Outcome Measure; SHAR: Swedish Hip Arthroplasty Register; THR: Total Hip Replacement.

## Competing interests

MG has no competing interests.

AP has no competing interests.

SO is the director of the Danish Hip Arthroplasty Register.

GG is the director of the Swedish Hip Arthroplasty Register at the Centre of Registers in Region Västra Götaland.

ABP has no competing interests.

OR is partly employed by the Swedish Hip Arthroplasty Register at the Centre of Registers in Region Västra Götaland.

## Authors’ contributions

MG participated in the design of the study, prepared and merged the databases, carried out the statistical analyses, and contributed to drafting the manuscript. AP participated in the design of the study, managed ethical review board approval in Denmark, and revised the manuscript. SO participated in the design of the study and revised the manuscript. GG participated in the design of the study and was responsible for the acquisition of Swedish data. ABP participated in the design of the study, was responsible for the acquisition of Danish data and revised the manuscript. OR participated in the design of the study, managed ethical review board approval in Sweden and was responsible for drafting the manuscript. All authors read and approved the final manuscript.

## Pre-publication history

The pre-publication history for this paper can be accessed here:

http://www.biomedcentral.com/1471-2474/14/316/prepub
